# Body mass index impacts the lipid-lowing effects of statins in patients with type 2 diabetes

**DOI:** 10.3389/fcvm.2025.1493613

**Published:** 2025-02-12

**Authors:** Lulu Sun, Zhuo Wang, Bai Wang, Yuxiang Jia, Qidi Zhao, Jingjun Zhao, Xingtao Huang

**Affiliations:** ^1^ Department of Endocrinology, The Second Affiliated Hospital of Harbin Medical University, Harbin, China; ^2^ Department of Cardiology, The First Affiliated Hospital of Harbin Medical University, Harbin, China; ^3^ Department of Cardiology, The Fifth Affiliated Hospital of Sun Yat-Sen University, Zhuhai, China; ^4^ Department of Cardiology, The Second Affiliated Hospital of Harbin Medical University, Harbin, China

**Keywords:** body mass index, statins, type 2 diabetes mellitus, low-density lipoprotein cholesterol, lipid

## Abstract

**Background:**

Individuals diagnosed with type 2 diabetes mellitus (T2DM) commonly exhibit elevated lipid levels and an increased body mass index (BMI). The impact of BMI on the efficacy of statins in reducing lipid levels among diabetic patients remains uncertain. We aim to evaluate whether BMI will affect the lipid-lowing effects of statins in patients with T2DM.

**Methods:**

In this retrospective analysis, we screened T2DM patients who were prescribed statins and underwent a 1-year outpatient follow-up recorded in the electronic medical record system. Patients were stratified into three groups: normal weight (BMI < 24 kg/m), overweight (24 kg/m^2^ ≤ BMI < 28 kg/m^2^), and obese (BMI ≥ 28 kg/m^2^). Lipid levels were assessed at two time points, and multivariate logistic regression analysis was used to identify factors influencing the reduction of low-density lipoprotein-cholesterol (LDL-C) levels and lipid control achievement.

**Results:**

This study included 289 patients, with 82 in normal weigh, 143 in overweight, and 64 in obese. Overweight and obese patients were found to be younger than those with normal weight. Over the 1-year follow-up period, lipid levels decreased in all patients, with a significant reduction observed in LDL-C levels. Notably, obese patients experienced the highest reduction in LDL-C levels compared to the normal and overweight groups (normal weight group *Δ*LDL 0.78 ± 0.95 mmol/L, *p* < 0.001; overweight group *Δ*LDL 0.80 ± 0.88 mmol/L, *p* < 0.001; obese group *Δ*LDL 1.11 ± 0.82 mmol/L, *p* < 0.038). Obese patients exhibited a remarkable 42.02% reduction in LDL levels (normal 27.45%, overweight 30.64%). Multivariate logistic regression analysis indicated that achieving lipid control, defined as LDL < 2.6 mmol/L, was more likely in obese patients compared to those with normal weight [odds ratio [OR] 3.48, 95% confidence interval [CI]: 1.18, 10.21, *p* = 0.023].

**Conclusions:**

The effectiveness of statins in lowering lipid levels appears to be influenced by the patient's BMI in patients with T2DM. T2DM patients with high BMI may derive greater benefits, particularly in LDL reduction, from statin therapy.

## 1 Introduction

Individuals with type 2 diabetes (T2DM) exhibit a heightened prevalence of lipid abnormalities, contributing to an increased susceptibility to atherosclerotic cardiovascular disease (1). In addition, patients with diabetes are usually associated with a higher body mass index (BMI), and more complicated metabolic profile compared to non-diabetic people (2), including altered levels of adipocytokines (such as adiponectin), hyperglycemia, and insulin resistance which are thought to be associated with dyslipidemia (3).

Statin therapy is currently the first-line treatment for diabetic individuals to prevent coronary artery disease (CAD) according to the recent recommendations from the American Heart Association (AHA) and the American Diabetes Association (ADA) (4, 5). A higher BMI may influence the efficacy of statins in lowering lipid levels. The *Reversal of Atherosclerosis with Aggressive Lipid Lowering* (REVERSAL) study demonstrated that participants with a baseline BMI below the median experienced the most significant proportional reduction in low-density lipoprotein cholesterol (LDL-C) (6). However, a study by Rachel A. Sharpton and colleagues found that obesity did not impact the lipid-lowering effectiveness of simvastatin therapy (7).Therefore, continued research into the effect of obesity on the efficacy of medications is essential. The metabolic condition of obesity combined with T2DM is more complex, and the therapeutic effects of statins in these patients remain unclear.

Our study's primary objective is to assess variations in the lipid-lowering outcomes of statin therapy among patients with T2DM who have different BMI levels

## 2 Material and methods

### 2.1 Study population

This research is an observational, retrospective study. We conducted a retrospective screening of patients with T2DM who were prescribed statins through the electronic medical record system of the Second Affiliated Hospital of Harbin Medical University from January 2022 to December 2022. The inclusion criteria for this study were as follows: (1) 18 ≤ age ≤ 80 years, (2) a confirmed diagnosis of T2DM, and (3) no prior use of lipid-lowering medications, (4) absence of any exclusion criteria.

The exclusion criteria were as follows: (1) combination therapy with non-statin lipid-lowering drugs, (2) liver and kidney dysfunction (liver dysfunction defined as alanine aminotransferase >40 U/L; kidney dysfunction defined as serum creatinine >40 μmol), (3) heart failure [defined as patients with New York Heart Association (NYHA) class II–IV symptoms or a documented left ventricular ejection fraction (LVEF) < 50%], (4) confirmed diagnosis of coronary artery disease [a history of coronarycomputed tomography angiography (CTA) or coronary angiography indicating coronary artery stenosis greater than 50%, or the presence of typical angina symptoms], (4) history of myocardial infarction, or coronary artery stent implantation, (5) changes in statin dosage or type during follow-up due to various reasons, (6) lack of 1-year follow-up data.

The ethics committee of The Second Affiliated Hospital of Harbin Medical University gave its approval to the research strategy.

### 2.2 Data collection and study definitions

Fasting serum glucose concentration of ≥7.0 mmol/L or 120 min postprandial plasma glucose (PPG) ≥ 11.1 mmol/L was defined as T2DM. Patients were divided into 3 groups based on their BMI level: normal weight (BMI < 24 kg/m^2^), overweight (24 kg/m^2^ ≤ BMI < 28 kg/m^2^), and obese (BMI ≥ 28 kg/m^2^) according to Chinese definition (8). All patients were treated with moderate-intensity statins (mainly atorvastatin 20 mg/d or rosuvastatin 10 mg/d) according to the recommendations of the guideline (9, 10), and the goal of lipid-lowering was that LDL-C < 2.6 mmol/L. The clinical data including sex, age, BMI, blood pressure, previous hypertension, smoking history and the level of lipid (two-time points) were collected. Clinical biochemical parameters were automatically tested using a fully automated biochemical analyzer (Roche Diagnostics cobas® c 702 module, USA). Changes in cholesterol levels between baseline and follow-up are expressed as the difference, represented by *Δ*TC, *Δ*TG, *Δ*HDL, and *Δ*LDL, respectively.

### 2.3 Statistical analyses

Categorical variables were presented as numbers and percentages, whereas continuous variables were shown as mean and standard deviation (normally distributed data) or median and interquartile range (non-normally distributed data). The *χ*^2^ test was used to compare categorical variables. The normality of data distribution was assessed using the Shapiro–Wilk test. Depending on the distribution, Student's *t*-tests or Wilcoxon rank-sum tests were used to compare continuous variables. A paired t-test was used to compare changes in cholesterol levels between baseline and follow-up for different groups.

Clinical outcomes were assessed using univariate logistic regression analysis. IBM SPSS Statistics (V.23, IBM Corp., Armonk, New York, USA) was used for all analyses. A *p*-value < 0.05 was considered statistically significant.

## 3 Results

### 3.1 Baseline characteristics

**Figure 1:**
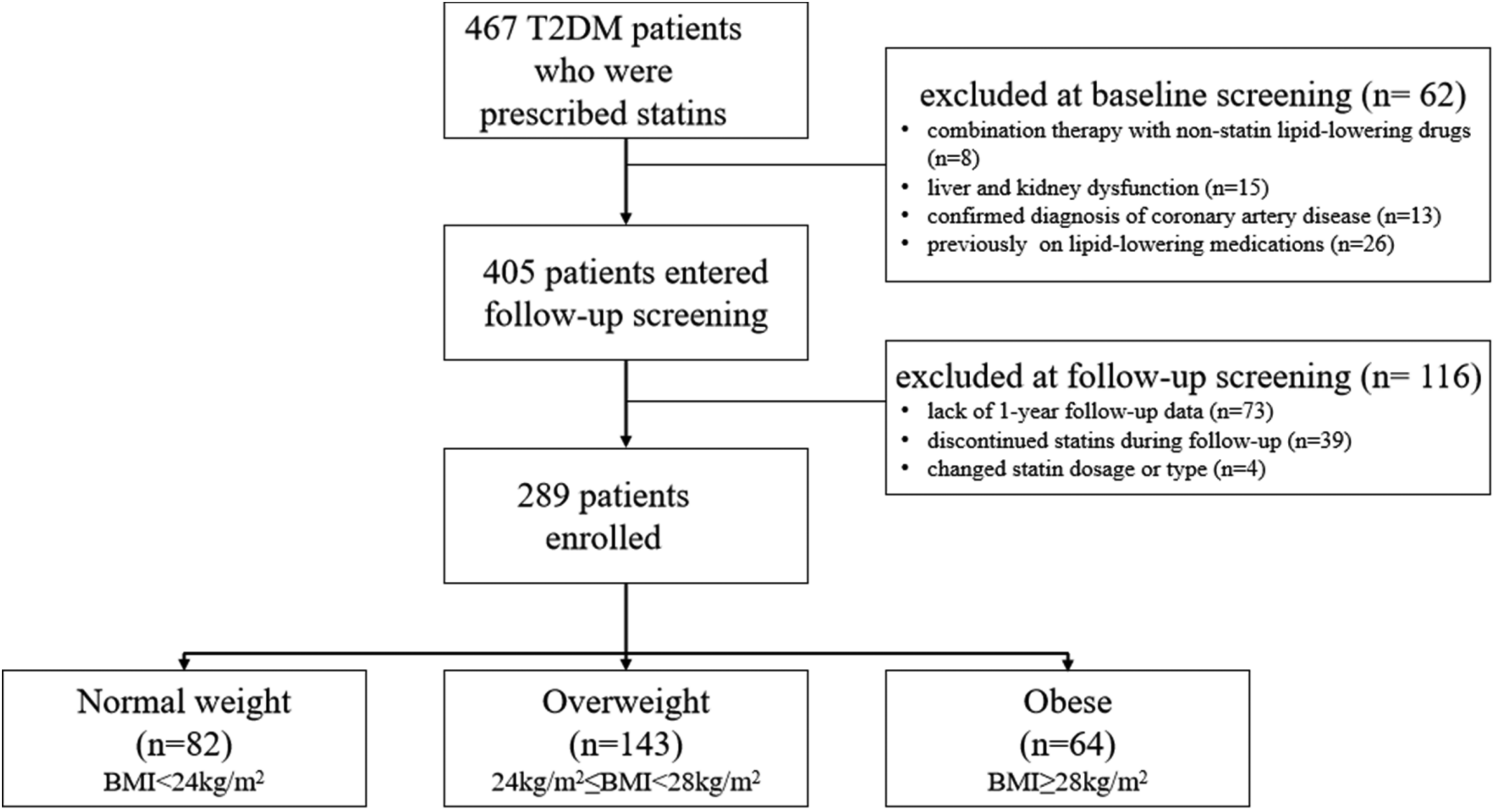
Flow diagram.

A total of 467 T2DM patients who were prescribed statins at the hospital were screened between January 1, 2022, and December 31, 2022. According to the inclusion and exclusion criteria, 178 individuals were excluded, and finally 289 patients were included in this study (Figure 1). The baseline characteristics are shown in Table 1.

**Table 1:** Characteristics of the patients at baseline.

	Normal *N* = 82	Overweight *N* = 143	Obese *N* = 64	*p*
BMI, kg/m^2^	22.11 ± 1.42	25.83 ± 1.18	30.50 ± 2.47	<0.001
Age, years.	65.23 ± 9.64	61.08 ± 10.33	59.80 ± 10.79	0.002
Male, *n* (%)	37 (45.1%)	116 (81.1%)	44 (68.8%)	<0.001
Hypertension, *n* (%)	47 (57.3%)	82 (57.3%)	47 (73.4%)	0.066
Current smoking, *n* (%)	18 (22.0%)	49 (34.3%)	21 (32.8%)	0.139
Total cholesterol, mmol/L	4.64 ± 1.19	4.57 ± 1.02	4.66 ± 1.10	0.768
Triacylglycerol, mmol/L	1.88 ± 1.31	2.01 ± 1.73	2.35 ± 2.05	0.218
HDL, mmol/L	1.32 ± 0.60	1.31 ± 0.64	1.15 ± 0.52	0.155
LDL, mmol/L	2.77 ± 1.02	2.71 ± 0.94	2.78 ± 0.90	0.784
LDL < 2.6 mmol/L, *n* (%)	34 (41.5%)	69 (48.3%)	28 (43.8%)	0.591
LDL < 1.8 mmol/L, *n* (%)	13 (15.9%)	22 (15.4%)	7 (10.9%)	0.649
FPG, mmol/L	11.39 ± 3.66	11.56 ± 4.11	11.03 ± 3.84	0.684
H1Abc, %	8.44 ± 1.55	8.26 ± 1.66	8.15 ± 1.40	0.835
Systolic pressure, mmHg	135.80 ± 23.89	136.35 ± 25.57	139.22 ± 23.72	0.673
Diastolic pressure, mmHg	77.33 ± 14.20	81.55 ± 14.01	81.64 ± 13.41	0.067
Statins				
Atorvastatin (20 mg), *n* (%)	63 (76.8%)	110 (76.9%)	52 (81.3%)	0.920
Rosuvastatin(10 mg), *n* (%)	12 (14.6%)	19 (13.3%)	8 (12.5%)	
Others, *n* (%)	7 (8.5%)	14(9.8%)	4(6.3%)	

BMI, body mass index; HDL, high-density lipoprotein-cholesterol; LDL, low-density lipoprotein-cholesterol; FPG, fasting plasma glucose; H1Abc, Hemoglobin A1c.

The BMI of the obese group was 30.50 ± 2.47 kg/m^2^[(mean ± standard deviation), *p* < 0.001], while the BMI of normal weight and overweight groups were 22.11 ± 1.42 kg/m^2^ (*p* < 0.001) and 25.83 ± 1.18 kg/m^2^ (*p* < 0.001), respectively. There are significantly different in sex and age among the three groups. Overweight and obese patients were younger than the normal-weight patients. Male patients were more likely to have higher BMI (81.1% in the overweight group, 68.8% in the obese group and only 45.1% in the normal-weight group). There were no significant differences in the prevalence of hypertension, current smoking, fasting plasma glucose, and hemoglobin A1c among the three groups.

In terms of lipid components, there were no significant statistical differences in total cholesterol (TC), triglycerides (TG), high-density lipoprotein (HDL), or LDL-C among the three groups. Most patients were treated with atorvastatin or rosuvastatin according to guideline recommendations. Although no statistically significant difference in medication choice were observed between the groups (*p* = 0.920), atorvastatin was the predominant option (Table 1).

The study population consisted of 467 T2DM patients who were prescribed statins at the hospital and screened between January 1, 2022 and December 31, 2022. Sixty-two patients were exclude at baseline screening due to combination therapy with non-statin lipid-lowering drugs (*n* = 8), liver or kidney dysfunction (*n* = 15), confirmed coronary artery disease (*n* = 13), and prior use of lipid-lowering medications (*n* = 26). A further 116 patients were exclude at follow-up screening due to lack of 1-year follow-up data (*n* = 73), discontinuation of statin therapy (*n* = 39), or changes in statin dosage or type (*n* = 4). In total, 289 patients were included in the study and divided into three groups based on BMI level: normal weight (BMI < 24 kg/m^2^, *n* = 82), overweight (24 kg/m^2^ ≤ BMI < 28 kg/m^2^, *n* = 143), and obese (BMI ≥ 28 kg/m^2^, *n* = 64).

### 3.2 The lipid-lowing effects of statins among different groups

**Figure 2:**
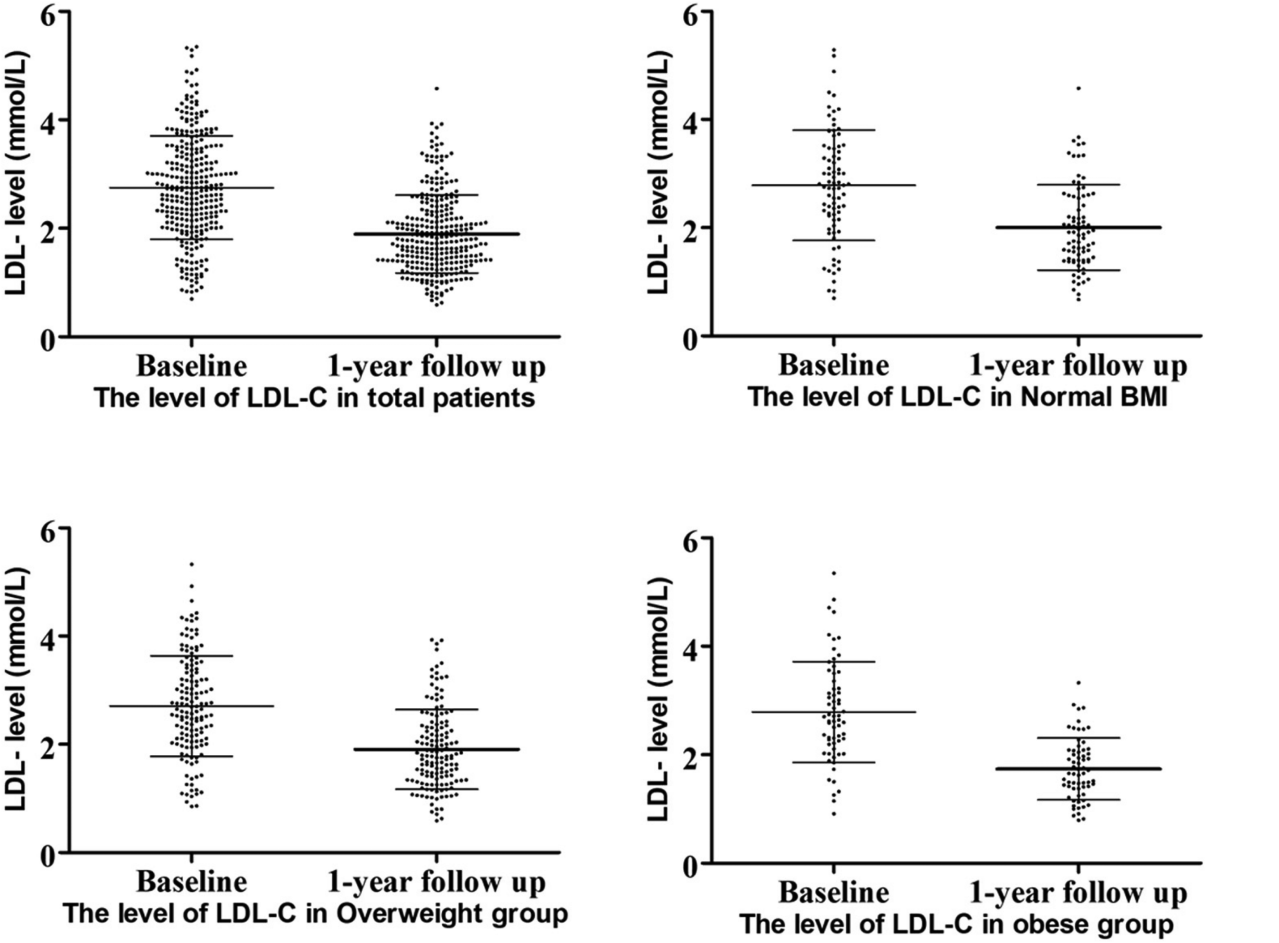
LDL-C levels at baseline and follow-up.

Lipid level decreased in all patients at the 1-year follow-up, with LDL-C levels showing the most significant reduction (Figure 2). All three groups exhibited decreases in TC and LDL-C levels: TC, normal weight group *Δ*TC 0.93 ± 1.29 mmol/L, *p* < 0.001; overweight group *Δ*TC 0.99 ± 1.08 mmol/L, *p* < 0.001; obese group *Δ*TC 1.26 ± 1.09 mmol/L, *p* < 0.001, and LDL-C, normal weight group *Δ*LDL 0.78 ± 0.95 mmol/L, *p* < 0.001; overweight group *Δ*LDL 0.80 ± 0.88 mmol/L, *p* < 0.001; obese group *Δ*LDL 1.11 ± 0.82 mmol/L, *p* < 0.001.

**Figure 3:**
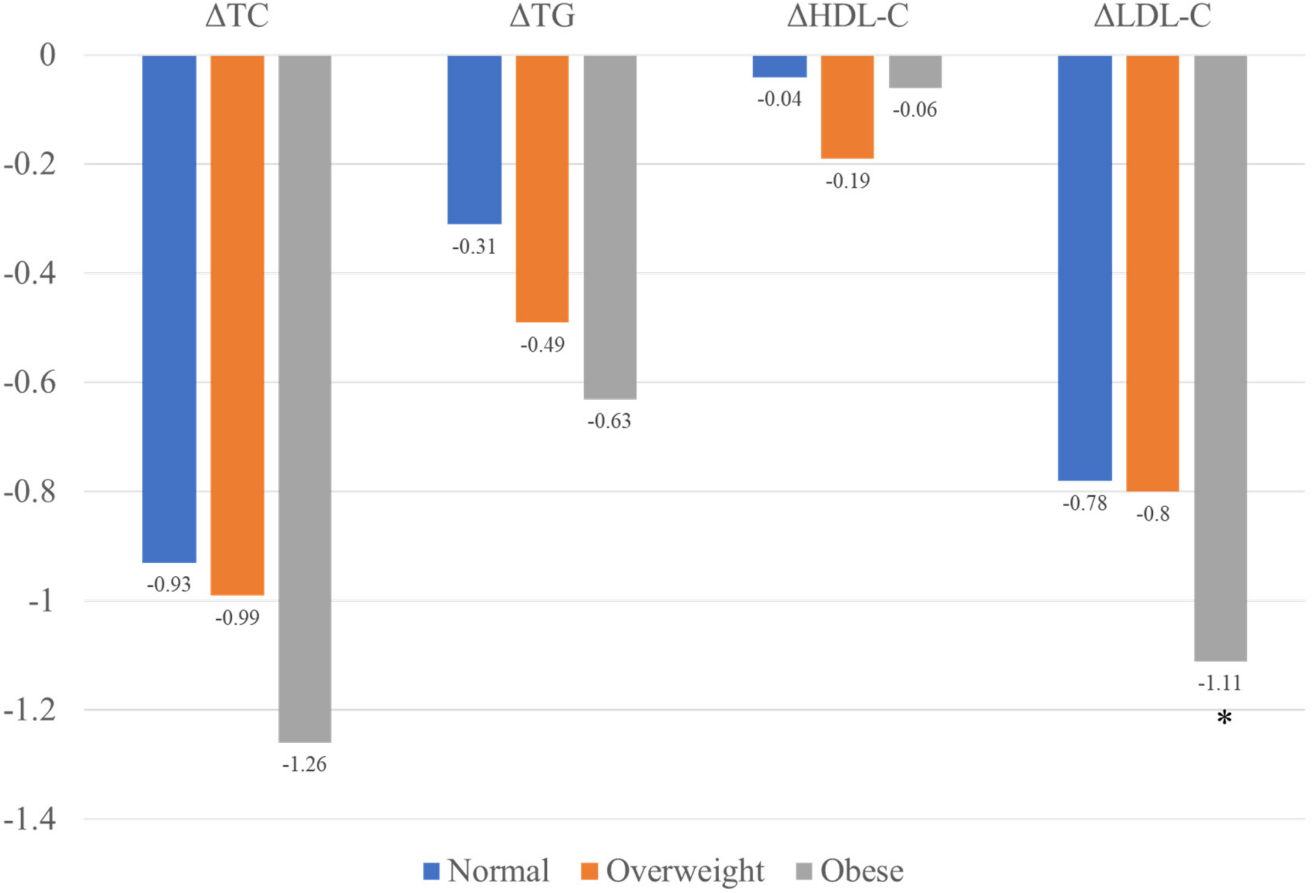
Changes in lipid levels among the three groups.

At the 1-year follow-up, no significant differences were observed in cholesterol levels (including LDL-C, TC, TG, and HDL) among the three groups. However, obese patients demonstrated the highest reduction in LDL-C levels compared to the other groups (Figure 3). A similar trend was observed for *Δ*LDL%,. with obese patientsshowing the highest reduction of 42.02% in the LDL-C levles.

Although the reduction in TC and TG levels were also higher in obese patients compared to normal-weight and overweight patients, these differences were no statistically significant. The lipid characteristic at follow-up are summarized in Table 2.

**Table 2:** The lipid characteristic of patients with different BMI at follow-up.

	Normal *N* = 82	Overweight *N* = 143	Obese *N* = 64	*p*
TC, mmol/L	3.71 ± 0.97	3.56 ± 0.88	3.39 ± 0.76	0.123
TG, mmol/L	1.56 ± 1.06	1.54 ± 0.73	1.71 ± 1.01	0.465
HDL-C, mmol/L	1.28 ± 0.62	1.11 ± 0.31	1.04 ± 0.25	0.002
LDL-C, mmol/L	2.00 ± 0.79	1.91 ± 0.74	1.74 ± 0.57	0.085
FPG, mmol/L	8.09 ± 2.82	8.19 ± 2.99	8.07 ± 2.72	0.957
H1Abc, %	7.64 ± 1.72	7.57 ± 1.54	7.39 ± 1.09	0.690
*Δ*TC, mmol/L	0.93 ± 1.29	0.99 ± 1.08	1.26 ± 1.09	0.212
*Δ*TG, mmol/L	0.31 ± 1.48	0.49 ± 1.66	0.63 ± 1.95	0.531
*Δ*HDL, mmol/L	0.04 ± 0.76	0.19 ± 0.51	0.06 ± 0.29	0.122
*Δ*LDL	0.78 ± 0.95	0.80 ± 0.88	1.11 ± 0.82	0.038
*Δ*LDL%	27.45 (7.07, 43.82)	30.64 (9.44, 46.88)	42.04 (21.55, 51.88)	0.010
*Δ*LDL% ≥ 50%, *n*	10 (12.2%)	24 (16.8%)	14 (21.9%)	0.296
LDL < 2.6 mmol/L	63 (76.8%)	119 (83.2%)	59 (92.2%)	0.047
LDL < 1.8 mmol/L	38 (46.3%)	74 (51.7%)	37 (57.8%)	0.387

TC, total cholesterol, TG, triacylglycerol, HDL, high-density lipoprotein-cholesterol; LDL, low-density lipoprotein-cholesterol; *Δ*TC (TG, HDL, LDL) means the level of TC (TG, HDL, LDL) at baseline minus the level of TC (TG, HDL, LDL) at follow-up. *Δ*LDL% means the level of LDL at baseline divided into *Δ*LDL multiplied by 100.

### 3.3 The relationship between BMI and control rate of lipid-lowering

Univariate logistic regression analysis indicated that obesity was associated with a higher lipid-lowering control rate compared to normal weight when the goal was defined as LDL < 2.6 mmol/L [odds ratio [OR] 3.56, 95% confidence interval [CI]: 1.25, 10.14, *p* = 0.018].After adjusting for sex, age, smokingn status, and hypertension, the association remained significant (OR 3.48, 95% CI: 1.18, 10.21, *p* = 0.023).

## 4 Discussion

In this study, we evaluated the effects of BMI on the lipid-lowering efficacy of statins. The results showed that obese patients experienced a significantly greater reduction in LDL-C levels one year after being prescribed statins. Further analysis revealed that obesity facilitated achieving the target lipid-lowering goal when patients were prescribed statins of the same intensity.

The relationship among statins, T2DM, and BMI is complex. Obesity plays a causal role in the development of type 2 diabetes (11) may mediate the diabetogenic effects of statins (12, 13). However, it remains unclear whether obesity influences the lipid-lowering efficacy of statins. Given that diabetes is often associated with obesity, it is of great clinical significance to investigate the impact of obesity on the effects of statins in this population. Previous studies have demonstrated that statin therapy increases the risk of new-onset type 2 diabetes (14), Furthermore, a Mendelian randomization study revealed that elevated BMI accounts for more than half of the diabetogenic effects associated with statin use (13). Despite these risks, statins effectively lower cholesterol and reduce cardiovascular risk in diabetic patients. Concerns remain regarding the potential of statins to worsen glucose control, as some studies suggest their use is associated with a small increase in hemoglobin A1c levels, reflecting a slight elevation in blood glucose (15). Nonetheless, the cardiovascular benefits of statins are widely regarded as outweighing these adverse effects on glycemic control (15). A large, real-world cohort study shows that BMI also influences the choice of lipid-lowering treatment, with statin intensity increasing as BMI rises (16). In our study, diabetic patients were treated with moderate-intensity statins according to the guidelines, but statin intensity was not adjusted based on BMI. This may lead to an underestimation of the lipid-lowering effects of statins in diabetic patients. However, this limitation does not affect our evaluation of the impact of BMI on the lipid-lowering efficacy of statins.

The Reversal of Atherosclerosis with Aggressive Lipid Lowering (REVERSAL) study showed that participants with a baseline BMI below the median exhibited the greatest proportional decrease in LDL-C (6), which contrasts with the findings of our study. In our study, obese patients demonstrated a more significant reduction in LDL-C levels one year after starting statin therapy.

It is important to note that in the REVERSAL study, the lipid-lowering effects varied between atorvastatin and pravastatin, with atorvastatin showing significantly better results in obese patients. The reasons for the paradoxical influence of obesity on statin efficacy remain uncertain. Some researchers speculate that the differential non-lipid-lowering properties of these agents, such as their anti-inflammatory effects, may explain this outcome. Therefore, our findings are not entirely unexpected.

The patients included in our study were diabetic, presenting with more complex metabolic abnormalities. These abnormalities, caused by obesity and hyperglycemia, may have affected the lipid-lowering efficacy of statins, leading to differences between obese and non-obese patients. Although patients with higher BMI achieved better lipid-lowering effects and were more likely to meet treatment goals after 12 months of statin therapy, the underlying reasons for these differences in statin efficacy remain uncertain.

We propose several theories regarding the impact of BMI on statin effectiveness. First, this effect may be related to the different apolipoprotein E (apoE) phenotypes commonly found in obese patients. Previous research has shown that individuals with simple obesity and diabetes are more likely to express the E2 phenotype compared to normal-weight patients (17). This genotype is more responsive to statins, resulting in the most significant reduction in blood lipids (18, 19), followed by the E3 type, while the E4 type has a lesser impact. E2 is particularly sensitive to statins in terms of reducing HDL-C levels, which aligns with the results of our study. Obese patients had the lowest HDL values after treatment.

Furthermore, obesity is often associated with changes in body composition and function, which can alter the pharmacokinetic and pharmacodynamic properties of many drugs (20). For example, obese individuals frequently experience pathological changes in the liver and kidneys, such as liver cirrhosis and chronic kidney disease, leading to impaired drug clearance (21, 22). Additionally, the cytochrome P450 (CYP) enzyme system plays a crucial role in the metabolism of statins (23). Obese individuals tend to have lower CYP3A4 expression and activity compared to normal-weight patients (24), which may result in a longer half-life for statins, thereby enhancing their lipid-lowering effects in obese individuals.

In our study, we observed a significant reduction in LDL-c levels across all BMI groups, with the greatest reduction observed in obese patients. However, total cholesterol did not show a comparable reduction, which is consistent with the mechanism of action of statins. Statins primarily target LDL-C, and while they effectively lower LDL-C levels, they do not always produce substantial changes in total cholesterol levels. This is because total cholesterol is composed of various lipid fractions, including LDL-C, high-density lipoprotein cholesterol, and very low-density lipoprotein cholesterol (VLDL-C). While statins are highly effective at reducing LDL-C, their effects on HDL-c and VLDL-c are less pronounced, which may explain why we did not observe a significant change in total cholesterol despite the reduction in LDL-C.

This study has several limitations. First, this is a retrospective single-center study, and the sample size is relatively small, which may introduce statistical bias. Second, changes in BMI over time were not evaluated, and fluctuations in BMI during the follow-up period may have influenced the results. Thirdly, we were unable to assess the patients’ dietary and lifestyle habits, which could have influenced their blood lipid levels and potentially impacted the lipid-lowering effectiveness of statins. Additionally, the patient's blood glucose control may influence the effectiveness of lipid management.Therefore, the absence of information on treatments for type 2 diabetes in our study could potentially affect the lipid-lowering efficacy of statins, leading to possible bias in our results. Fourth, patients who adjusted or changed their statin dosage or type during the follow-up period were excluded to minimize confounding factors. However, this approach might overestimate the efficacy of statins. Nevertheless, this overestimation is unlikely to affect the evaluation of the relationship between BMI and statin efficacy. Finally, we did not include a comparison with non-diabetic patients, leaving it unclear whether the observed phenomenon also occurs in non-diabetic individuals. Future studies with larger sample sizes and comparative designs are needed to further explore whether BMI influences the efficacy of statins in the general population.

## 5 Conclusion

Patients with T2DM are often accompanied by obesity and dyslipidemia. Statins play a critical role in the primary prevention of CAD in T2DM patients.The effectiveness of statins in lowering lipid levels appears to be influenced by the patient's BMI in patients with T2DM. T2DM patients with high BMI may derive greater benefits, particularly in LDL reduction, from statin therapy.

## Data Availability

The raw data supporting the conclusions of this article will be made available by the authors, without undue reservation.

## References

[1] American Diabetes Association Professional Practice Committee. 10. Cardiovascular disease and risk management: standards of medical care in diabetes—2021. Diabetes Care. (2021) 47:S179–218. 10.2337/dc24-S010 PMC10725811 38078592

[2] Chan DC Watts GF. Dyslipidaemia in the metabolic syndrome and type 2 diabetes: pathogenesis, priorities, pharmacotherapies. Expert Opin Pharmacother. (2011) 12:13–30. 10.1517/14656566.2010.502529 20629587

[3] Verges B. Pathophysiology of diabetic dyslipidaemia: where are we? Diabetologia. (2015) 58:886–99. 10.1007/s00125-015-3525-8 25725623 PMC4392164

[4] Arnett DK Blumenthal RS Albert MA Buroker AB Goldberger ZD Hahn EJ 2019 ACC/AHA guideline on the primary prevention of cardiovascular disease: executive summary: a report of the American College of Cardiology/American Heart Association task force on clinical practice guidelines. Circulation. (2019) 140:e563–95. 10.1161/CIR.0000000000000677 30879339 PMC8351755

[5] Doyle-Delgado K Chamberlain JJ Shubrook JH Skolnik N Trujillo J. Pharmacologic approaches to glycemic treatment of type 2 diabetes: synopsis of the 2020 American diabetes association's standards of medical care in diabetes clinical guideline. Ann Intern Med. (2020) 173:813–21. 10.7326/M20-2470 32866414

[6] Nicholls SJ Tuzcu EM Sipahi I Schoenhagen P Hazen SL Ntanios F Effects of obesity on lipid-lowering, anti-inflammatory, and antiatherosclerotic benefits of atorvastatin or pravastatin in patients with coronary artery disease (from the REVERSAL study). Am J Cardiol. (2006) 97:1553–7. 10.1016/j.amjcard.2005.12.042 16728212

[7] Sharpton R Laucka P McKeller R Dangler M Horne J Dangler J. The impact of obesity on the efficacy of simvastatin for lowering low-density lipoprotein cholesterol in a veteran population. Fed Pract. (2017) 34:41–4. 30766266 PMC6370421

[8] Powell-Wiley TM Poirier P Burke LE Despres JP Gordon-Larsen P Lavie CJ Obesity and cardiovascular disease: a scientific statement from the American Heart Association. Circulation. (2021) 143:e984–1010. 10.1161/CIR.0000000000000973 33882682 PMC8493650

[9] Grundy SM Stone NJ Bailey AL Beam C Birtcher KK Blumenthal RS 2018 AHA/ACC/AACVPR/AAPA/ABC/ACPM/ADA/AGS/APhA/ASPC/NLA/PCNA guideline on the management of blood cholesterol: a report of the American College of Cardiology/American Heart Association task force on clinical practice guidelines. Circulation. (2019) 139:e1082–143. 10.1161/CIR.0000000000000625 30586774 PMC7403606

[10] Xiao Y Yu B Chao C Wang S Hu D Wu C Chinese expert consensus on blood lipid management in patients with diabetes (2024 edition). J Transl Int Med. (2024) 12:325–43. 10.2478/jtim-2024-0014 39360162 PMC11444477

[11] Censin JC Peters SAE Bovijn J Ferreira T Pulit SL Mägi R Causal relationships between obesity and the leading causes of death in women and men. PLos Genet. (2019) 15:e1008405. 10.1371/journal.pgen.1008405 31647808 PMC6812754

[12] Wu PT Moon JY Daghlas I Franco G Porneala BC Ahmadizar F Obesity partially mediates the diabetogenic effect of lowering LDL cholesterol. Diabetes Care. (2022) 45:232–40. 10.2337/dc21-1284 34789503 PMC8753762

[13] Yang GY Schooling CM. Statins, type 2 diabetes, and body mass index: a univariable and multivariable Mendelian randomization study. J Clin Endocr Metab. (2023) 108:385–96. 10.1210/clinem/dgac562 36184662

[14] Swerdlow DI Preiss D Kuchenbaecker KB Holmes MV Engmann JE Shah T HMG-coenzyme A reductase inhibition, type 2 diabetes, and bodyweight: evidence from genetic analysis and randomised trials. Lancet. (2015) 385:351–61. 10.1016/S0140-6736(14)61183-1 25262344 PMC4322187

[15] Hoogwerf BJ. Statins may increase diabetes, but benefit still outweighs risk. Cleve Clin J Med. (2023) 90:53–62. 10.3949/ccjm.90a.22069 36596598

[16] Ferrieres J Lautsch D Gitt AK De Ferrari G Toplak H Elisaf M Body mass index impacts the choice of lipid-lowering treatment with no correlation to blood cholesterol—findings from 52,916 patients in the dyslipidemia international study (DYSIS). Diabetes Obes Metab. (2018) 20:2670–4. 10.1111/dom.13415 29888459 PMC6220851

[17] Galal AA Abd Elmajeed AA Elbaz RA Wafa AM Elshazli RM. Association of apolipoprotein E gene polymorphism with the risk of T2DM and obesity among Egyptian subjects. Gene. (2021) 769:145223. 10.1016/j.gene.2020.145223 33059023

[18] Wang Y Du X Zhao R Niu J Wang H Li J. Association of APOE polymorphisms with lipid-lowering efficacy of statins in atherosclerotic cardiovascular diseases. Ann Acad Med Singap. (2021) 50:474–80. 10.47102/annals-acadmedsg.2020505 34195754

[19] Konialis C Spengos K Iliopoulos P Karapanou S Gialafos E Hagnefelt B The APOE E4 allele confers increased risk of ischemic stroke among Greek carriers. Adv Clin Exp Med. (2016) 25:471–8. 10.17219/acem/38841 27629735

[20] Utrup TR Mueller EW Healy DP Callcut RA Peterson JD Hurford WE. High-dose ciprofloxacin for serious gram-negative infection in an obese, critically ill patient receiving continuous venovenous hemodiafiltration. Ann Pharmacother. (2010) 44:1660–4. 10.1345/aph.1P234 20736424

[21] Ratziu V Giral P Charlotte F Bruckert E Thibault V Theodorou I Liver fibrosis in overweight patients. Gastroenterology. (2000) 118:1117–23. 10.1016/S0016-5085(00)70364-7 10833486

[22] Guzzaloni G Grugni G Minocci A Moro D Morabito F. Liver steatosis in juvenile obesity: correlations with lipid profile, hepatic biochemical parameters and glycemic and insulinemic responses to an oral glucose tolerance test. Int J Obes Relat Metab Disord. (2000) 24:772–6. 10.1038/sj.ijo.0801224 10878685

[23] Hirota T Fujita Y Ieiri I. An updated review of pharmacokinetic drug interactions and pharmacogenetics of statins. Expert Opin Drug Metab Toxicol. (2020) 16:809–22. 10.1080/17425255.2020.1801634 32729746

[24] Sankaralingam S Kim RB Padwal RS. The impact of obesity on the pharmacology of medications used for cardiovascular risk factor control. Can J Cardiol. (2015) 31:167–76. 10.1016/j.cjca.2014.10.025 25661551

